# Comparison of Long-Term Outcomes and Associated Factors between Younger and Older Rural Ischemic Stroke Patients

**DOI:** 10.3390/jcm11051430

**Published:** 2022-03-05

**Authors:** Durgesh Chaudhary, Michelle Anyaehie, Francis Demiraj, Shreya Bavishi, Shima Shahjouei, Jiang Li, Vida Abedi, Ramin Zand

**Affiliations:** 1 Department of Neurology, Neuroscience Institute, Geisinger Health System, Danville, PA 17822, USA; dpchaudhary@geisinger.edu (D.C.); sshimashah@gmail.com (S.S.); 2 Department of Medical Education, Geisinger Commonwealth School of Medicine, Scranton, PA 18510, USA; manyaehie@som.geisinger.edu; 3 Dr. Kiran C. Patel College of Osteopathic Medicine, Nova Southeastern University, Davie, FL 33314, USA; fd341@mynsu.nova.edu; 4 Cell and Molecular Biology Department, Tulane University, New Orleans, LA 70118, USA; bavishi.shreya@gmail.com; 5 Department of Molecular and Functional Genomics, Weis Center for Research, Geisinger Health System, Danville, PA 17822, USA; jli@geisinger.edu; 6 Department of Public Health Sciences, College of Medicine, The Pennsylvania State University, Hershey, PA 17033, USA; vidaabedi@gmail.com; 7 Neuroscience Institute, The Pennsylvania State University, Hershey, PA 17033, USA

**Keywords:** young stroke, ischemic stroke, all-cause mortality, ischemic stroke recurrence, long-term outcomes, long-term survival

## Abstract

Introduction: The rise of ischemic stroke among young adults has stressed the need to understand their risk profiles and outcomes better. This study aimed to examine the five-year ischemic stroke recurrence and survival probability among young patients in rural Pennsylvania. Methods: This retrospective cohort study included first-time ischemic stroke patients from the Geisinger Health System between September 2003 and May 2014. The outcomes included all-cause mortality and ischemic stroke recurrence at five years. Kaplan-Meier estimator, cumulative incidence function, Cox proportional hazards model, and Cause-specific hazard model were used to examine the association of independent variables with the outcomes. Results: A total of 4459 first-time ischemic stroke patients were included in the study, with 664 (14.9%) patients in the 18–55 age group and 3795 (85.1%) patients in the >55 age group. In the 18–55 age group, the five-year survival probability was 87.2%, and the cumulative incidence of recurrence was 8%. Patients in the 18–55 age group had significantly lower hazard for all-cause mortality (HR = 0.37, 95% CI 0.29–0.46, *p* < 0.001), and non-significant hazard for five-year recurrence (HR = 0.81, 95% CI 0.58–1.12, *p* = 0.193) compared to the >55 age group. Chronic kidney disease was found to be associated with increased mortality in the 18–55 age group. Conclusion: In our rural population, younger ischemic stroke patients were at the same risk of long-term ischemic stroke recurrence as the older ischemic stroke patients. Identifying the factors and optimizing adequate long-term secondary prevention may reduce the risk of poor outcomes among younger ischemic stroke patients.

## 1. Introduction

While the overall prevalence of stroke has decreased, there has been a relative increase in the hospitalization rate of young adults with stroke in the past decade [1,2,3,4]. The reported mortality rates in young stroke patients from different studies have been varied, ranging from around 10% at 5 years to more than 20% at 20 years [5,6,7,8]. These studies have shown lower mortality in the young ischemic stroke patients compared to the elderly but much higher than in the general population [5,6,7,8]. Malignancy, heart failure, heavy drinking, type 1 diabetes, and large artery atherosclerosis were found to be independent predictors of long-term mortality in young ischemic stroke patients [5]. Studies on long-term recurrence risk among young stroke patients are relatively scarce. However, 10% of young stroke patients are estimated to have a recurrence within five years of the first episode [9]. Varona et al. reported a recurrence rate of 25% in a cohort of ischemic stroke patients aged 15 to 45 years with a mean follow-up of 12.3 years [8]. There is also a paucity of studies on the long-term outcomes among young stroke patients in the rural United States.

There is no uniform definition of young stroke, and the upper age cut-off has most commonly varied from 45 to 55 years in recent literature [5,6,7,8,10,11]. This variation in age cut-off is another factor that makes a comparison between different studies difficult. In this study, the age cut-off for young stroke was set at 55 years. The young ischemic stroke patients were further divided into subgroups to be in line with the age cut-off set by other studies.

The additional knowledge regarding the long-term outcomes and associated factors among the young rural stroke population can help with targeted and measurable interventions. The American Heart Association has highlighted the need for more data in this cohort in order to create treatment recommendations in the future [12].

This study aimed at examining the long-term ischemic stroke recurrence and survival probability among young patients within a hospital-based cohort and comparing it to older ischemic stroke patients in rural Pennsylvania.

## 2. Materials and Methods

### 2.1. Data Source

This retrospective observational study utilized the ischemic stroke database from Geisinger Health System named Geisinger NeuroScience Ischemic Stroke (GNSIS). GNSIS is a database of 8929 ischemic stroke patients from September 2003 to May 2019. Patients were included in the GNSIS database if they had a primary diagnosis of ischemic stroke based on International Classification of Diseases, Ninth/Tenth Revision, Clinical Modification (ICD-9/10-CM) codes, had brain magnetic resonance imaging (MRI) in the same encounter, and had at least one overnight stay at the hospital. GNSIS contains longitudinal clinical data for ischemic stroke patients compiled from various sources, including electronic health records data (EHR), social security death data, and quality data. The GNSIS database has been described in detail in previous studies [13,14,15]. The study was reviewed and approved by the Geisinger Institutional Review Board.

### 2.2. Cohort Definition and Outcome Measures

Our cohort consisted of patients from the GNSIS database, with age of at least 18 years, and an index stroke date between 3 September 2003 and 22 May 2014. Patients were excluded if they had a prior history of ischemic or hemorrhagic stroke. Patients were divided into two subgroups based on their age at index stroke— “18–55” and “above 55” age group.

**Figure 1:**
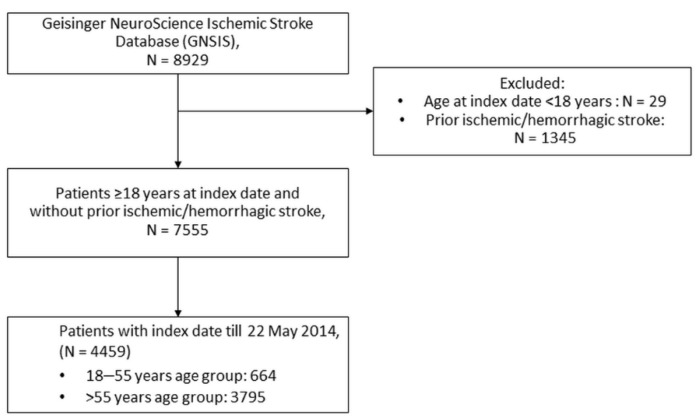
Inclusion and exclusion flowchart of patients in the study.

Patients with index stroke date after 22 May 2014 were excluded as a five-year follow-up would not be complete for these patients on 22 May 2019, and their inclusion could result in biased estimates in the survival analysis [16]. The patient inclusion/exclusion flow chart is shown in Figure 1.

As there are different reported age cut-offs for young stroke in the various studies, the “18–55” age group were further divided into “18–49” and “49–55” age groups in subgroup analysis I (based on median age in “18–55” group) and into “18–43.7” and “43.7–55” age group in subgroup analysis II (based on the first quartile value of age).

The outcomes were ischemic stroke recurrence and all-cause mortality within five years of the stroke date (index stroke). Ischemic stroke recurrence was defined from the EHR using ICD-9/10-CM code in the primary diagnosis along with MRI brain in the same encounter. All-cause mortality was assessed from the EHR data and cross-referenced with the social security death database on 22 May 2019. Thus, the follow-up time was defined as the time between the index stroke date and the last encounter in the EHR for recurrence and between the index stroke date and the end of the study period (22 May 2019) for the all-cause mortality.

### 2.3. Statistical Analysis

All categorical variables were summarized as count and percentage and all continuous variables as mean ± standard deviation (SD) or median with interquartile range (IQR). Differences between subgroups were examined using Pearson’s chi-square test or Fisher’s exact test for categorical variables, and the Mann-Whitney U test for continuous variables.

The Kaplan–Meier estimator was used to estimate survival probability at various time points, and the log-rank test was used to examine the difference in survival curves of different groups. A stratified Cox proportional hazards model was used to investigate the factors related to all-cause mortality. The proportional hazards assumptions of the Cox model were tested using the Schoenfeld residuals test.

For ischemic stroke recurrence, a competing risk analysis was performed in which all-cause mortality was included as a competing outcome. It is observed that all-cause mortality is a competing outcome against ischemic stroke recurrence in the long term. In such cases, the Kaplan–Meier estimator (and its complement function) and Cox proportional hazards model can produce biased results, and competing risk analysis is recommended [17]. The cumulative incidence function was used to estimate the incidence of ischemic stroke recurrence. The cause-specific hazard model was used to investigate the factors associated with ischemic stroke recurrence. The Cox model and cause-specific hazard model results were expressed as hazard ratio (HR) with 95% confidence interval (CI), an estimate of the ratio of the hazard rate in a group versus the control group. For all analyses, *p* < 0.05 was considered statistically significant. All analyses were done in R version 4.0.3.

## 3. Results

### 3.1. Patient Demographics and Stroke Risk Factors

There were 4459 first-time ischemic stroke patients included in the study, with 664 (14.9%) patients aged 18 to 55 years at the time of stroke and 3795 (85.1%) patients were 55 years or older. Table 1 includes the demographics and clinical characteristics of the study cohorts.

Among the 664 patients in the 18–55 age group, 394 (59.3%) were men and had a median age of 49 [interquartile range (IQR) 43.7–52.4] years compared to 50.4% men in the >55 age group with a median age of 74.4 [IQR 65.8–82.0] years. In the 18–55 age group, the most common comorbidity was hypertension (52.1%), followed by dyslipidemia (42.6%) and diabetes (24.7%). The rates of most comorbidities in the younger group were significantly lower than in the >55 groups (Table 1). However, in the 18–55 age group, 128 (19.3%) had a patent foramen ovale (PFO), and 22 (3.3%) had a hypercoagulable state diagnosis which was significantly higher than in the >55 age group. The median National Institutes of Health Stroke Scale (NIHSS) was 3 [IQR 1-6] in the 18–55 age group compared to 4 [IQR 2–7] in the >55 age group. Due to the high rate of missingness of NIHSS, it was excluded from further analysis.

### 3.2. All-Cause Mortality within 5 Years of Index Stroke in Young Stroke Patients

In the five years following the index stroke, 84 (12.7%) patients in the 18–55 age group were deceased. The deceased young patients had significantly higher rates of hypertension, diabetes, congestive heart failure, myocardial infarction, peripheral vascular disease, chronic kidney disease, and a lower rate of PFO (Table 2). Comparison between the alive and deceased patients for the entire cohort is provided in Supplemental Table S1.

**Figure 2:**
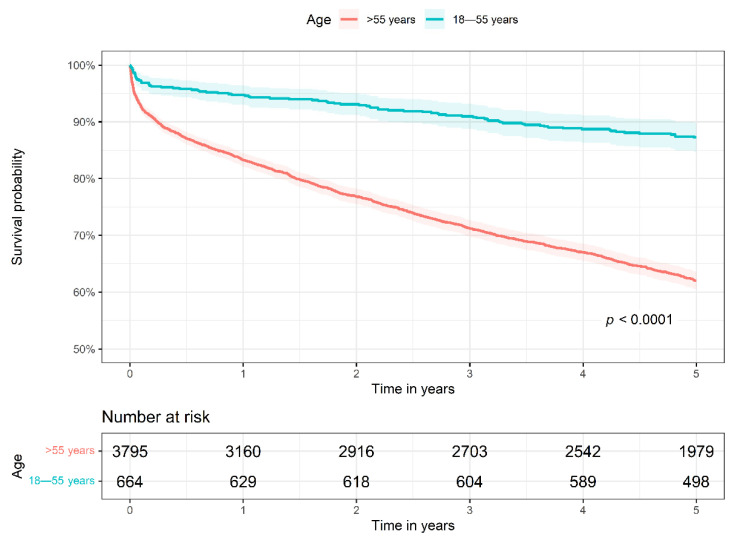
Comparison of survival probabilities between 18–55 age group and >55 age group using Kaplan-Meier estimator.

Using the Kaplan–Meier estimator, the survival probability was significantly higher for the 18–55 age group than the >55 age group (log-rank test *p* < 0.0001, Figure 2). For the 18–55 age group, the survival probability was found to be 94.7% (95% confidence interval [CI] 93.0–96.4), 91.0% (95% CI 88.8–93.2), and 87.2% (95% CI 84.7–89.8) at 1 year, 3 years and 5 years, respectively. The >55 age group had a lower survival probability of 83.3% (95% CI 82.1–84.5), 71.2% (95% CI 69.8–72.7), and 62.0% (95% CI 60.5–63.6) at 1 year, 3 years and 5 years, respectively.

Three stratified Cox models were built to examine the outcome of five-year all-cause mortality. The first Cox model (CoxPH1) included all patients and age as a binary variable with >55 years as the reference. In CoxPH1, the 18–55 age group was found to have a significantly lower hazard for five-year all-cause mortality (HR = 0.37, 95% CI 0.29–0.46, *p* < 0.001). The second Cox model (CoxPH2) included all patients with age as a continuous variable. In CoxPH2, age was found to be significantly associated with five-year all-cause mortality (HR = 1.04, 75% CI 1.04–1.05, *p* < 0.001).

The third Cox model (CoxPH3) only included patients in the 18–55 age group to examine the factors associated with five-year all-cause mortality in this age group. In CoxPH3, chronic kidney disease was found to be significantly associated with an increased hazard of all-cause mortality in the 18–55 age group (HR = 3.38, 95% CI 1.73–6.62, *p* < 0.001). The details of the three Cox models are provided in Supplemental Tables S2–S4.

### 3.3. Ischemic Stroke Recurrence within Five Years of Index Stroke in Young Stroke Patients

Among the patients in the 18–55 age group, 384 patients were found to have five years of follow-up or ischemic stroke recurrence during this period. Of these 384 patients, 46 (12%) had ischemic stroke recurrence in the five years following the index stroke date. There was no significant difference in terms of gender between the recurrent and non-recurrent groups. The patients with ischemic stroke recurrence had significantly higher rates of congestive heart failure (8.7% vs. 2.4% in the non-recurrent group, *p* = 0.043). The patients with recurrence also had a higher proportion of current and former smokers, but more concrete conclusions could not be made due to a high number of patients with unknown smoking status. Supplemental Table S1 includes the comparison between the recurrent and non-recurrent groups for the entire cohort.

**Figure 3:**
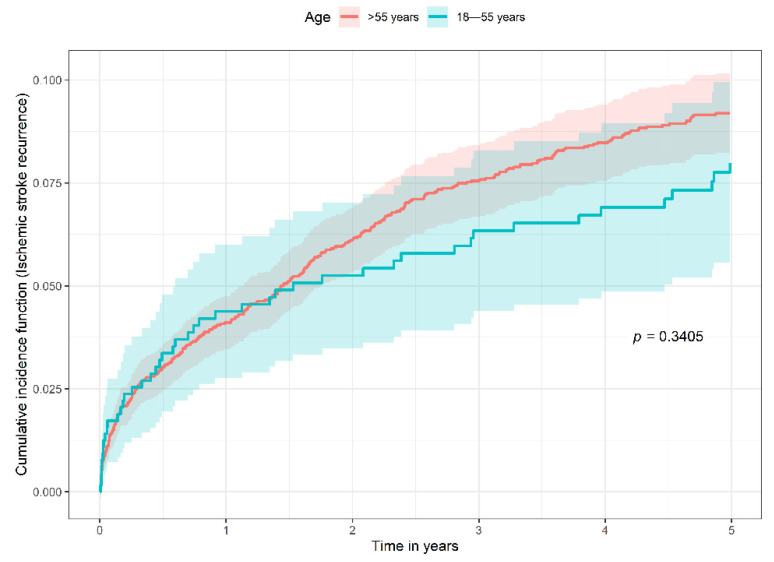
Cumulative incidence function of ischemic stroke recurrence in the 18–55 age group compared to >55 age group (competing risk of mortality not shown).

The cumulative incidence of ischemic stroke recurrence among the 18–55 age group was not significantly different from that of the >55 age group (Gray’s test, *p* = 0.3405, Figure 3). The cumulative incidence of ischemic stroke recurrence in the 18–55 age group was 4.4% (95% CI 2.8–6.0), 6.3% (95% CI 4.4–8.3), and 8.0% (95% CI 5.8–10.2) at 1 year, 3 years and 5 years, respectively. In comparison, the cumulative incidence of ischemic stroke recurrence in the >55 age group was 4.1% (95% CI 3.5–4.8), 7.6% (95% CI 6.7–8.4), and 9.2 (95% CI 8.2–10.2) at 1 year, 3 years and 5 years, respectively.

Three cause-specific hazard models (CSHM) were built for the outcome of five-year ischemic stroke recurrence. In CSHM1, all patients were included, and age was included as a binary variable with >55 years as the reference. In CSHM1, the 18–55 age group had non-significant hazard for recurrence compared to the >55 age group (HR = 0.81, 95% CI 0.58–1.12, *p* = 0.193). In CSHM2, all patients were included, and age was used as a continuous variable. In CSHM2, age was found to be significantly associated with recurrence (HR = 1.01, 95% CI 1.001–1.02, *p* = 0.025). In the CSHM3, only patients in the 18–55 age group were included, and age was not found to be significant (HR = 0.99, 95% CI 0.96–1.04, *p* = 0.98). There was a trend toward significance for congestive heart failure between the recurrent and non-recurrent group in CSHM3 (HR = 2.77, 9%% CI 0.98–7.82, *p* = 0.054). The details of the three cause-specific hazard models are presented in the supplemental Tables S5–S7.

### 3.4. Subgroup Analyses

Two subgroup analyses were performed by dividing the 18–55 age group into two subgroups based on the median and first quartile value of age.

**Figure 4:**
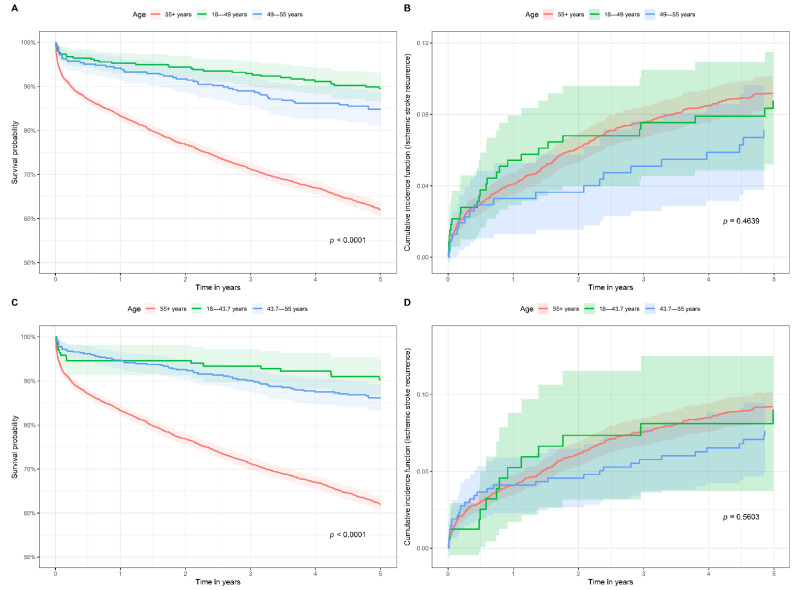
(**A**) Kaplan–Meier survival curves for 18–49, 49–55, and 55+ age groups. (**B**) Cumulative incidence of ischemic stroke recurrence for 18–49, 49–55, and 55+ age groups. (**C**) Kaplan–Meier survival curves for 18–43.7, 43.7–55, and 55+ age groups. (**D**) Cumulative incidence of ischemic stroke recurrence for 18–43.7, 43.7–55, and 55+ age groups.

In subgroup analysis I, the 18–55 age group was divided into the 18–49 age group and the 49–55 age group. The survival probability was not significantly different between the 18–49 age group and the 49–55 age group but survival was significantly higher in these two age groups compared to the >55 age group (Figure 4A). In the Cox model, the 18–49 age group (HR = 0.30, 95% CI 0.21–0.41, *p* < 0.001) and the 49–55 age group (HR = 0.44, 95% CI 0.33–0.59, *p* < 0.001) had significantly lower hazard for five-year all-cause mortality compared to the >55 age group. The cumulative incidence of ischemic stroke recurrence was similar in the 18–49, 49–55, and >55 age groups (Figure 4B). In the cause-specific hazard model, the 18–49 age group (HR = 0.90, 95% CI 0.59–1.38, *p* = 0.631) and the 49–55 age group (HR = 0.71, 95% CI 0.45–1.13, *p* = 0.148) had non-significant hazard for recurrence compared to the >55 age group.

The 18–55 age group was divided into the 18–43.7 age group and the 43.7–55 age group in subgroup analysis II. Similar to the results in subgroup analysis I, the survival probability was not significantly different between the 18–43.7 age group and the 43.7–55 age group, but survival was significantly higher in these two age groups compared to the >55 age group (Figure 4C). In the Cox model, the 18–43.7 age group (HR = 0.27, 95% CI 0.16–0.44, *p* < 0.001) and the 43.7–55 age group (HR = 0.40, 95% CI 0.31–0.51, *p* < 0.001) had significantly lower hazard for five-year all-cause mortality compared to the >55 age group. The cumulative incidence of ischemic stroke recurrence was similar in the 18–43.7, 43.7–55, and >55 age groups (Figure 4D). In the cause-specific hazard model, the 18–43.7 age group (HR = 0.92, 95% CI 0.52–1.63, *p* = 0.784) and the 43.7–55 age group (HR = 0.77, 95% CI 0.53–1.11, *p* = 0.164) had non-significant hazard for recurrence compared to the >55 age group.

The clinical characteristics of the subgroups in the two subgroup analyses are given in Supplemental Tables S8 and S9.

## 4. Discussion

Our study examined the long-term outcomes in young stroke patients in a rural and predominantly Caucasian population. Our study showed similar five-year ischemic stroke recurrence rates in the younger and older age groups (8% and 9.2% in the younger and older age group, respectively) and significantly lower five-year all-cause mortality in the younger age group (12.8% and 38% in the younger and older age group respectively). To our knowledge, this is the first study on the long-term recurrence and all-cause mortality of young ischemic stroke patients from a rural population.

A review study by Varona et al. [18] showed cumulative mortality rates increasing as years progressed from an initial ischemic stroke in young patients. A 4.9% increase in cumulative mortality rate was seen for the first year and about 4% in the following 2 to 5 years [18]. Our study showed similar results with around 5% cumulative mortality in year 1 and almost 13% at 5 years. A study [19] in Canada which included a sample size of 1341 patients, reported 97.8% (year 1), 95.3% (year 2), and 92.9% (year 3) survival probability among young patients (<45 years) with ischemic stroke or transient ischemic attack which was similar to the survival probabilities reported in our study (95% in the first year and 91% in the third year). Higher mortality rates were seen for older patients within our study, comparable to other population-based studies on young stroke. [6,7,20,21] In addition, our study showed similar rates of ischemic stroke recurrence, as noted by Smajlovic et al. [9]

In our study, the 49–55 age group (in subgroup analysis I) and the 43.7–55 age group (in subgroup analysis II) had significantly higher rates of hypertension, dyslipidemia, diabetes, and peripheral vascular disease compared to their younger counterparts but lower than in the >55 age group (Supplemental Tables S8 and S9). These patients, especially the 49–55 age group, represent a mixed group of patients with or without the common risk factors for ischemic stroke. In our Cox regression analysis, age, chronic kidney disease, congestive heart failure, atrial fibrillation, and neoplasm were significant factors in all-cause mortality for the entire cohort (CoxPH1, Supplemental Table S2). Still, chronic kidney disease was the only significant factor associated with mortality in our younger stroke patient cohort (CoxPH3, Supplemental Table S4). Diabetes and PFO were found to be significantly associated with ischemic stroke recurrence for the entire cohort (CSHM1 and CHSM2, Supplemental Tables S5 and S6).

Studies have shown key risk factors associated with stroke in younger patients, including dyslipidemia, hypertension, diabetes mellitus, and age [22]. PFO was also associated with young stroke in our study. This may be attributed to the level of imaging procedures and evaluations received at hospitals since younger patients essentially have fewer risk factors for stroke, and PFO is more likely to be investigated as a cause in younger adults. Interestingly, the patients who were alive at 5 years following a stroke had higher rates of PFO. Although we could not investigate this observation further, it may indicate that patients with PFO had better post-stroke evaluation and follow-ups.

Limitations of this study include a small proportion of young stroke patients in comparison to the entire cohort. The definition of young stroke is not clear in the current literature. Some studies [9,23,24] consider young stroke as low as 15 years and as high as 59 years; other studies, including this study, find 18–55 years to be the best fit for the definition of young stroke. [25,26] The risk factors for ischemic stroke were ascertained at the time of hospitalization for ischemic stroke and changes in risk factors over time were not evaluated. Due to the study design and how patients were grouped, a minority of patients in the young stroke group turned 55 years over the course of follow-up. Another limitation of this study was that the effect of health care management on the outcomes was not included in the study. Moreover, NIHSS was missing at a high rate and could not be included in the multivariate analysis. Further studies are needed to define young stroke based on etiologies, risk factors, or outcomes.

## 5. Conclusions

In our rural population, the five-year risk of mortality and recurrence in young ischemic stroke patients was 12.8% and 8%, respectively. Younger ischemic stroke patients were at the same risk of long-term ischemic stroke recurrence as the older ischemic stroke patients. Identifying the factors and optimizing adequate long-term secondary prevention may reduce the risk of poor outcomes among younger ischemic stroke patients.

## Figures and Tables

**Table 1:** Demographics and clinical characteristics of patients in the study.

	Overall	18–55 Age Group	>55 Age Group	*p*
Number of patients, n	4459	664	3795	
Sex: Male, n (%)	2305 (51.7)	394 (59.3)	1911 (50.4)	<0.001
Age at index stroke in years, median [IQR]	71.5 [60.7, 80.8]	49.0 [43.7, 52.4]	74.4 [65.8, 82.0]	-
Hypertension, n (%)	3169 (71.1)	346 (52.1)	2823 (74.4)	<0.001
Atrial fibrillation, n (%)	814 (18.3)	14 (2.1)	800 (21.1)	<0.001
Dyslipidemia, n (%)	2555 (57.3)	283 (42.6)	2272 (59.9)	<0.001
Diabetes, n (%)	1338 (30.0)	164 (24.7)	1174 (30.9)	0.001
Congestive heart failure, n (%)	519 (11.6)	23 (3.5)	496 (13.1)	<0.001
Myocardial infarction, n (%)	473 (10.6)	41 (6.2)	432 (11.4)	<0.001
Peripheral vascular disease, n (%)	685 (15.4)	48 (7.2)	637 (16.8)	<0.001
Hypercoagulable state, n (%)	59 (1.3)	22 (3.3)	37 (1.0)	<0.001
Chronic kidney disease, n (%)	604 (13.5)	36 (5.4)	568 (15.0)	<0.001
Neoplasm, n (%)	684 (15.3)	23 (3.5)	661 (17.4)	<0.001
Rheumatic diseases, n (%)	165 (3.7)	12 (1.8)	153 (4.0)	0.007
Patent foramen ovale, n (%)	384 (8.6)	128 (19.3)	256 (6.7)	<0.001
Smoking status				<0.001
Current smoker, n (%)	563 (12.6)	170 (25.6)	393 (10.4)	
Former smoker, n (%)	956 (21.4)	72 (10.8)	884 (23.3)	
Never smoker, n (%)	1275 (28.6)	136 (20.5)	1139 (30.0)	
Unknown, n (%)	1665 (37.3)	286 (43.1)	1379 (36.3)	
NIHSS *, median [IQR]	4.0 [2.0, 7.0]	3.0 [1.0, 6.0]	4.0 [2.0, 7.0]	0.015

* NIHSS was only available for 557 patients overall (81 in 18–55 age group, 476 patients in >55 age group).

**Table 2:** Patient characteristics in the 18–55 age group stratified by five-year outcomes.

	All-Cause Mortality at 5 Years in the 18–55 Age Group			Ischemic Stroke Recurrence at 5 Years in the 18–55 Age Group		
	Alive	Deceased	*p*	No Recurrence	Recurrence	*p*
Number of patients, n	580	84		338	46	
Sex: Male, n (%)	341 (58.8)	53 (63.1)	0.528	193 (57.1)	24 (52.2)	0.636
Age at index stroke in years, median [IQR]	48.7 [43.5, 52.4]	49.9 [45.9, 52.3]	0.082	48.8 [43.4, 52.3]	47.7 [43.5, 51.8]	0.627
Hypertension, n (%)	293 (50.5)	53 (63.1)	0.041	164 (48.5)	21 (45.7)	0.835
Atrial fibrillation, n (%)	11 (1.9)	3 (3.6)	0.403	5 (1.5)	0 (0.0)	1.000
Dyslipidemia, n (%)	244 (42.1)	39 (46.4)	0.524	143 (42.3)	23 (50.0)	0.407
Diabetes, n (%)	129 (22.2)	35 (41.7)	<0.001	67 (19.8)	15 (32.6)	0.073
Congestive heart failure, n (%)	15 (2.6)	8 (9.5)	0.003	8 (2.4)	4 (8.7)	0.043
Myocardial infarction, n (%)	30 (5.2)	11 (13.1)	0.010	17 (5.0)	2 (4.3)	1.000
Peripheral vascular disease, n (%)	33 (5.7)	15 (17.9)	<0.001	22 (6.5)	5 (10.9)	0.349
Hypercoagulable state, n (%)	18 (3.1)	4 (4.8)	0.508	10 (3.0)	2 (4.3)	0.644
Chronic kidney disease, n (%)	20 (3.4)	16 (19.0)	<0.001	13 (3.8)	3 (6.5)	0.422
Neoplasm, n (%)	18 (3.1)	5 (6.0)	0.196	10 (3.0)	3 (6.5)	0.195
Rheumatic diseases, n (%)	10 (1.7)	2 (2.4)	0.656	6 (1.8)	1 (2.2)	0.594
Patent foramen ovale, n (%)	120 (20.7)	8 (9.5)	0.023	73 (21.6)	11 (23.9)	0.868
Smoking status			0.146			0.004
Current smoker, n (%)	145 (25.0)	25 (29.8)		92 (27.2)	16 (34.8)	
Former smoker, n (%)	58 (10.0)	14 (16.7)		37 (10.9)	6 (13.0)	
Never smoker, n (%)	123 (21.2)	13 (15.5)		69 (20.4)	17 (37.0)	
Unknown, n (%)	254 (43.8)	32 (38.1)		140 (41.4)	7 (15.2)	
NIHSS, median [IQR]	3.0 [1.0, 6.0]	2.0 [1.0, 3.5]	0.666	4.0 [1.5, 7.0]	3.0 [2.0, 3.8]	0.654

## Data Availability

The data analyzed in this study is not publicly available due to privacy and security concerns. The data may be made available after completing a data-sharing agreement with Geisinger Health System.

## Supplementary Materials

The following supporting information can be downloaded at: https://www.mdpi.com/article/10.3390/jcm11051430/s1, Table S1: Clinical characteristics of the entire cohort stratified by the outcomes; Table S2: Cox Model 1 (CoxPH1) for all patients (age as categorical); Table S3: Cox Model 2 (CoxPH2) for all patients (age as numeric); Table S4: Cox Model 3 (CoxPH3) for 18–55 age group patients (age as numeric); Table S5: CSHM Model 1 (CSHM1) for all patients (age as categorical); Table S6: CSHM Model 2 (CSHM2) for all patients (Age as numeric); Table S7: CSHM Model 3 (CSHM3) for 18–55 years age group (age as numeric). Table S8: Clinical characteristics of patients in the 18-49 age group versus 49-55 age group; Table S9. Clinical characteristics of patients in the 18-43.7 age group versus 43.7-55 age group.
