# Supplementary material for: Comparison of Long-Term Outcomes and Associated Factors between Younger and Older Rural Ischemic Stroke Patients
Source: J Clin Med. 2022 Mar 5;11(5):1430. doi: 10.3390/jcm11051430 (PMC8911514; doi:10.3390/jcm11051430)
Supplement: Supplementary file 1 [file jcm-11-01430-s001.zip › jcm-1571728-supplementary.pdf]

SUPPLEMENTAL MATERIAL

For

"Comparison of Long-term Outcomes and Associated Factors Between Younger and Older Rural Ischemic Stroke Patients"

Table of Contents:

Table S1. Clinical characteristics of the entire cohort stratified by the outcomes.

Table S2. Cox Model 1: All patients (age as categorical)

Table S3. Cox Model 2: all patients (age as numeric)

Table S4: Cox Model 3: 18-55 age group patients (age as numeric)

Table S5. CSHM Model 1: for recurrence for all patients (age as categorical)

Table S6. CSHM Model 2: for recurrence for all patients (Age as numeric)

Table S7. CSHM Model 3: for 18-55 years age group (age as numeric)

Table S8. Clinical characteristics of patients in the 18-49 age group versus 49-55 age group

Table S9. Clinical characteristics of patients in the 18-43.7 age group versus 43.7-55 age group

Table S1. Clinical characteristics of the entire cohort stratified by the outcomes.

	**All-cause mortality at 5 years among patients with at least 5 years of follow-up**			**Ischemic stroke recurrence at 5 years among patients with at least 5-year follow-up**		
	**Alive**	**Deceased**	**p**	**No recurrence**	**Recurrence**	**p**
**Number of patients, n**	2949	1510		1653	363	
**Sex: Male, n (%)**	1543 (52.3)	762 (50.5)	0.253	871 (52.7)	169 (46.6)	0.039
**Age at index stroke in years, median [IQR]**	67.8 [57.7, 77.4]	78.5 [69.1, 84.5]	<0.001	67.2 [57.4, 75.9]	71.9 [62.2, 80.7]	<0.001
**Hypertension, n (%)**	2078 (70.5)	1091 (72.3)	0.226	1149 (69.5)	272 (74.9)	0.047
**Atrial fibrillation, n (%)**	430 (14.6)	384 (25.4)	<0.001	230 (13.9)	73 (20.1)	0.004
**Dyslipidemia, n (%)**	1771 (60.1)	784 (51.9)	<0.001	993 (60.1)	235 (64.7)	0.112
**Diabetes, n (%)**	817 (27.7)	521 (34.5)	<0.001	438 (26.5)	147 (40.5)	<0.001
**Congestive heart failure, n (%)**	204 (6.9)	315 (20.9)	<0.001	103 (6.2)	50 (13.8)	<0.001
**Myocardial infarction, n (%)**	272 (9.2)	201 (13.3)	<0.001	158 (9.6)	44 (12.1)	0.169
**Peripheral vascular disease, n (%)**	393 (13.3)	292 (19.3)	<0.001	219 (13.2)	70 (19.3)	0.004
**Hypercoagulable state, n (%)**	37 (1.3)	22 (1.5)	0.674	24 (1.5)	4 (1.1)	0.805
**Chronic kidney disease, n (%)**	271 (9.2)	333 (22.1)	<0.001	141 (8.5)	57 (15.7)	<0.001
**Neoplasm, n (%)**	343 (11.6)	341 (22.6)	<0.001	211 (12.8)	61 (16.8)	0.051
**Rheumatic diseases, n (%)**	93 (3.2)	72 (4.8)	0.009	53 (3.2)	18 (5.0)	0.138
**Patent foramen ovale, n (%)**	303 (10.3)	81 (5.4)	<0.001	184 (11.1)	44 (12.1)	0.654
**Smoking status, n (%)**			<0.001			<0.001
**Current smoker, n (%)**	393 (13.3)	170 (11.3)		232 (14.0)	45 (12.4)	
**Former smoker, n (%)**	591 (20.0)	365 (24.2)		377 (22.8)	85 (23.4)	
**Never smoker, n (%)**	811 (27.5)	464 (30.7)		468 (28.3)	168 (46.3)	
**Unknown, n (%)**	1154 (39.1)	511 (33.8)		576 (34.8)	65 (17.9)	
**NIHSS, median [IQR]**	4.0 [2.0, 6.0]	4.0 [2.0, 8.0]	0.039	4.0 [2.0, 7.0]	3.0 [2.0, 5.0]	0.377

Table S2. Cox Model 1: All patients (age as categorical)

Term	HR	95% CI	p
Gender: Male	0.96	0.87 - 1.06	0.43
**Age group: 18-55**	**0.37**	**0.29 - 0.46**	**<0.001**
Atrial fibrillation	1.27	1.12 - 1.43	<0.001
Congestive heart failure	1.84	1.60 - 2.11	<0.001
Myocardial infarction	1.08	0.92 - 1.27	0.328
Chronic kidney disease	1.69	1.48 - 1.93	<0.001
Neoplasm	1.57	1.39 - 1.78	<0.001
Rheumatic disease	1.24	0.98 - 1.58	0.08

Table S3. Cox Model 2: all patients (age as numeric)

Term	HR	95% CI	p
**Age**	**1.04**	**1.04 - 1.05**	**<0.001**
Atrial fibrillation	1.09	0.96 - 1.23	0.173
Congestive heart failure	1.77	1.54 - 2.04	<0.001
Myocardial infarction	1.09	0.93 - 1.28	0.273
Chronic kidney disease	1.52	1.33 - 1.73	<0.001
Neoplasm	1.34	1.18 - 1.52	<0.001
Rheumatic disease	1.24	0.97 - 1.58	0.086

Table S4: Cox Model 3: 18-55 age group patients (age as numeric)

Term	HR	95% CI	p
Gender: Male	1.09	0.70 - 1.72	0.695
**Age**	**1.04**	**0.99 - 1.07**	**0.069**
Hypertension	0.83	0.49 - 1.42	0.501
Diabetes	1.38	0.80 - 2.37	0.25
Congestive heart failure	1.92	0.88 - 4.19	0.101
Myocardial infarction	1.64	0.83 - 3.26	0.156
Chronic kidney disease	3.38	1.73 - 6.62	<0.001

Table S5. CSHM Model1: for recurrence for all patients (age as categorical)

Term	HR	95% CI	p
Gender: Male	0.79	0.64 - 0.97	0.022
**Age group: 18-55**	**0.81**	**0.58 - 1.12**	**0.193**
Hypertension	0.93	0.71 - 1.22	0.609
Dyslipidemia	1.05	0.83 - 1.33	0.689
Diabetes	1.58	1.26 - 1.98	<0.001
Congestive heart failure	1.26	0.92 - 1.73	0.158
Peripheral vascular disease	1.23	0.94 - 1.60	0.137
Neoplasm	1.11	0.84 - 1.47	0.458
PFO	1.49	1.08 - 2.05	0.016

Table S6. CSHM Model2: for recurrence for all patients (age as numeric)

Term	HR	95% CI	p
Gender: Male	0.80	0.65 - 0.98	0.034
**Age**	**1.01**	**1.001 - 1.02**	**0.025**
Dyslipidemia	1.03	0.81 - 1.31	0.797
Diabetes	1.60	1.27 - 2.01	<0.001
Congestive heart failure	1.26	0.92 - 1.74	0.152
Peripheral vascular disease	1.23	0.94 - 1.61	0.133
Neoplasm	1.07	0.81 - 1.43	0.626
PFO	1.55	1.12 - 2.14	0.008

Table S7. CSHM Model3: for 18-55 years age group (age as numeric)

Term	HR	95% CI	p
Gender: Male	0.75	0.42 - 1.34	0.331
**Age**	**0.99**	**0.96 - 1.04**	**0.977**
Diabetes	1.47	0.78 - 2.79	0.233
Congestive heart failure	2.77	0.98 - 7.82	0.054

Table S8. Clinical characteristics of patients in the 18-49 age group versus 49-55 age group

	**18-49 Age group**	**49-55 Age group**	**p**
**Number of patients, n**	338	326	
**Sex: Male, n (%)**	192 (56.8)	202 (62.0)	0.203
**Age at index stroke in years, median [IQR]**	43.9 [37.7, 47.0]	52.5 [50.8, 53.8]	<0.001
**Hypertension, n (%)**	140 (41.4)	206 (63.2)	<0.001
**Atrial fibrillation, n (%)**	7 (2.1)	7 (2.1)	1.000
**Dyslipidemia, n (%)**	115 (34.0)	168 (51.5)	<0.001
**Diabetes, n (%)**	65 (19.2)	99 (30.4)	0.001
**Congestive heart failure, n (%)**	12 (3.6)	11 (3.4)	1.000
**Myocardial infarction, n (%)**	17 (5.0)	24 (7.4)	0.259
**Peripheral vascular disease, n (%)**	14 (4.1)	34 (10.4)	0.002
**Hypercoagulable state, n (%)**	15 (4.4)	7 (2.1)	0.129
**Chronic kidney disease, n (%)**	20 (5.9)	16 (4.9)	0.610
**Neoplasm, n (%)**	10 (3.0)	13 (4.0)	0.528
**Rheumatic diseases, n (%)**	4 (1.2)	8 (2.5)	0.255
**Patent foramen ovale, n (%)**	85 (25.1)	43 (13.2)	<0.001
**Smoking status**			0.003
**Current smoker, n (%)**	82 (24.3)	88 (27.0)	
**Former smoker, n (%)**	24 (7.1)	48 (14.7)	
**Never smoker, n (%)**	81 (24.0)	55 (16.9)	
**Unknown, n (%)**	151 (44.7)	135 (41.4)	
**NIHSS, median [IQR]**	2.0 [1.0, 4.0]	3.0 [2.0, 6.0]	0.115

Table S9. Clinical characteristics of patients in the 18-43.7 age group versus 43.7-55 age group

	**18-43.7 Age group**	**43.7-55 Age group**	**p**
**Number of patients, n**	167	497	
**Sex: Male, n (%)**	92 (55.1)	302 (60.8)	0.230
**Age at index stroke in years, median [IQR]**	37.5 [32.2, 41.2]	50.7 [47.9, 53.1]	<0.001
**Hypertension, n (%)**	58 (34.7)	288 (57.9)	<0.001
**Atrial fibrillation, n (%)**	3 (1.8)	11 (2.2)	1.000
**Dyslipidemia, n (%)**	45 (26.9)	238 (47.9)	<0.001
**Diabetes, n (%)**	27 (16.2)	137 (27.6)	0.004
**Congestive heart failure, n (%)**	5 (3.0)	18 (3.6)	0.811
**Myocardial infarction, n (%)**	9 (5.4)	32 (6.4)	0.713
**Peripheral vascular disease, n (%)**	4 (2.4)	44 (8.9)	0.005
**Hypercoagulable state, n (%)**	7 (4.2)	15 (3.0)	0.458
**Chronic kidney disease, n (%)**	11 (6.6)	25 (5.0)	0.434
**Neoplasm, n (%)**	7 (4.2)	16 (3.2)	0.624
**Rheumatic diseases, n (%)**	3 (1.8)	9 (1.8)	1.000
**Patent foramen ovale, n (%)**	44 (26.3)	84 (16.9)	0.010
**Smoking status**			0.005
Current smoker, n (%)	36 (21.6)	134 (27.0)	
Former smoker, n (%)	12 (7.2)	60 (12.1)	
Never smoker, n (%)	49 (29.3)	87 (17.5)	
Unknown, n (%)	70 (41.9)	216 (43.5)	
**NIHSS, median [IQR]**	2.0 [1.0, 4.0]	3.0 [1.0, 6.0]	0.235
